# Grouped multi-scale vision transformer for medical image segmentation

**DOI:** 10.1038/s41598-025-95361-8

**Published:** 2025-04-01

**Authors:** Zexuan Ji, Zheng Chen, Xiao Ma

**Affiliations:** School of Computer Science and Engineering, Nanjing University of Science and Technology, Nanjing, 210094 China

**Keywords:** Medical image segmentation, Transformer, Self-attention, Channel group, Multi-scale, Image processing, Machine learning

## Abstract

Medical image segmentation plays a pivotal role in clinical diagnosis and pathological research by delineating regions of interest within medical images. While early approaches based on Convolutional Neural Networks (CNNs) have achieved significant success, their limited receptive field constrains their ability to capture long-range dependencies. Recent advances in Vision Transformers (ViTs) have demonstrated remarkable improvements by leveraging self-attention mechanisms. However, existing ViT-based segmentation models often struggle to effectively capture multi-scale variations within a single attention layer, limiting their capacity to model complex anatomical structures. To address this limitation, we propose Grouped Multi-Scale Attention (GMSA), which enhances multi-scale feature representation by grouping channels and performing self-attention at different scales within a single layer. Additionally, we introduce Inter-Scale Attention (ISA) to facilitate cross-scale feature fusion, further improving segmentation performance. Extensive experiments on the Synapse, ACDC, and ISIC2018 datasets demonstrate the effectiveness of our model, achieving state-of-the-art results in medical image segmentation. Our code is available at: https://github.com/Chen2zheng/ScaleFormer.

## Introduction

Medical image segmentation, as a cornerstone of medical image analysis, aims to precisely delineate anatomically and pathologically relevant regions for quantitative clinical assessment. This process serves as the critical foundation for computer-aided diagnosis systems, enabling data-driven decision-making through automated extraction of diagnostically salient biomarkers^1–4^.

**Fig. 1:**
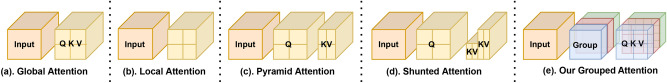
Comparing our grouped multi-scale attention with self-attention in Global, Local, Pyramid and Shunted.

Early convolutional architectures like U-Net^5^ and CE-Net^6^ established initial benchmarks through local feature extraction. However, their inherently limited receptive fields severely constrain global context modeling and long-range dependency learning^7–10^, resulting in suboptimal performance for complex anatomical structures. The transformer revolution in NLP sparked a paradigm shift in computer vision, culminating in ViT^11^—the first full-transformer architecture that pioneered image tokenization by splitting images into patch sequences (Fig. 1a). This token-based self-attention mechanism enables explicit global context capturing, overcoming convolutional constraints while achieving state-of-the-art performance. Building on this momentum, medical vision transformers including TransUnet^12^ and SwinUnet^13^ have established new benchmarks, demonstrating the transformative potential of systematic attention mechanism integration in medical image analysis.

While ViT-based approaches have demonstrated promising performance in medical image segmentation, their ability to handle multi-scale anatomical variations within a single attention layer remains fundamentally limited. As depicted in Fig. 1b, Local Attention mechanisms^14^ adopt non-overlapping window partitioning to reduce computational complexity. However, this single-scale strategy fails to adapt to the substantial size variations of anatomical structures in medical imaging, particularly when segmenting small lesions alongside large organs. Pyramid Attention^15^ attempts to address this through spatial token reduction (Fig. 1c), but its aggressive token aggregation in single-layer processing often causes critical small-object features to be subsumed by background noise^16^. A more sophisticated approach, Shunted Attention^16^ (Fig. 1d), introduces hierarchical downsampling of key-value pairs to capture multi-scale context. While improving scale adaptability, this design creates two inherent limitations: (1) Progressive downsampling inevitably discards high-frequency details crucial for precise boundary delineation, and (2) The static query formulation restricts cross-scale feature interaction. These shortcomings become particularly pronounced in medical imaging where millimeter-level precision is required for clinical diagnosis. Beyond scale limitations, conventional multi-head attention architectures face inherent expressiveness constraints. Recent analyses reveal significant redundancy among attention heads^17,18^, attributed to homogeneous input processing and insufficient inter-head communication^19^. Although cascaded attention mechanisms^19^ attempt to mitigate this through sequential head processing, they sacrifice the parallel computation advantage essential for efficient model training.

To address these challenges, we propose Grouped Multi-Scale Attention (GMSA), which simultaneously captures multi-scale features and enhances the expressiveness of self-attention, as illustrated in Fig. 1(e). Specifically, GMSA first groups input features along the channel dimension and then applies attention computations at different scales for each group to enhance the model’s ability to capture multi-scale features. Unlike Global, Local, Pyramid, and Shunted Attention, which either operate at a single-scale or suffer from information loss due to downsampling, GMSA preserves fine-grained details while enhancing feature diversity. In addition, to further expand the scope of multi-scale learning, we introduce the Inter-Scale Attention (ISA) mechanism. Applied after GMSA, ISA enables cross-scale feature fusion by integrating attention weight maps across different scales, which helps bridge the information gap between coarse- and fine-grained representations.

Consequently, the main contribution of this paper can be summarized as follows:

- Channel grouping for enhanced feature representation: We propose a channel grouping strategy where input features are divided into multiple groups, each independently generating Q/K/V. This design encourages diverse feature representations across groups, providing a structured way to capture different aspects of the input.
- Multi-scale attention within a single layer: Building on channel grouping, our Grouped Multi-Scale Attention (GMSA) applies self-attention at different scales within each group, allowing simultaneous multi-scale feature learning without additional layers.
- Inter-scale attention (ISA) for cross-scale fusion: We introduce an inter-scale attention (ISA) mechanism that enables cross-scale fusion by aggregating weight maps from different scales, facilitating multi-scale feature integration.

## Background

### Spatial dimension procedure in vision transformer

Current Vision Transformers employ two primary strategies for efficient feature extraction in the spatial dimension^14,15,20^: (1) partitioning the feature map into local regions and performing self-attention within each region or (2) merging tokens to reduce their overall number. A notable example of local self-attention is the Swin Transformer, which divides feature maps into non-overlapping square regions and applies self-attention within each segment. On the other hand, the Pyramid Vision Transformer (PVT) adopts spatial token reduction, merging tokens in the key and query branches to improve efficiency. However, local attention operates at a single scale, limiting its ability to capture multi-scale information, while spatial token merging, as used in PVT, can overly aggregate tokens, leading to the loss of fine-grained details-particularly for small structures that may blend into the background. These limitations make it challenging to segment organs of varying sizes and shapes, a common issue in medical image analysis. To tackle this, we propose a grouped multi-scale attention approach that computes attention for tokens at different scales, allowing the capture of feature information at various granularities.

### Channel dimension procedure in vision transformer

The multi-head self-attention (MHSA) mechanism inherently implements channel grouping by distributing features across parallel heads^21^. Each head projects distinct Q/K/V subspaces, followed by linear recombination through $1 \times 1$ convolutions. This design enables partial feature decoupling while maintaining model capacity.

However, efficient feature grouping does not necessarily mean full utilization of channel dimensions. Michel et al.^17^ analyzed the impact of removing individual attention heads and found that most were redundant-eliminating them had little effect on model performance. This redundancy was consistent across multiple domains. Liu et al.^19^ further investigated this issue by measuring cosine similarity between attention heads, confirming that redundancy arises because heads often receive highly similar feature inputs. To mitigate this, Liu et al. proposed a cascading mechanism, where each head receives progressively refined feature representations. While this improves attention diversity, it disrupts the parallelism of MHSA, leading to reduced computational efficiency. Instead, we propose a parallel feature grouping strategy combined with multi-scale attention mechanisms, which enhances feature diversity while preserving computational efficiency.

### Differences from existing ViT-based approaches

A key distinction between our approach and existing ViT-based methods is the explicit partitioning of the feature space before generating queries, keys, and values. Specifically, we first divide the feature space into multiple groups and generate independent sub-queries, sub-keys, and sub-values for each group. This strategy, similar to those in^18,19^, allows different groups to capture distinct feature representations, potentially enhancing feature diversity. However, unlike cascaded attention^19^ and single-head attention^18^, our method retains the multi-head attention mechanism while performing computations in parallel within each group. Furthermore, compared to traditional spatial ViT methods that rely on single-scale partitioning^14,20^ or global self-attention^11^, our Grouped Multi-Scale Attention (GMSA) introduces a multi-scale attention mechanism across different groups to enhance the representation capability of multi-scale features.

**Fig. 2:**
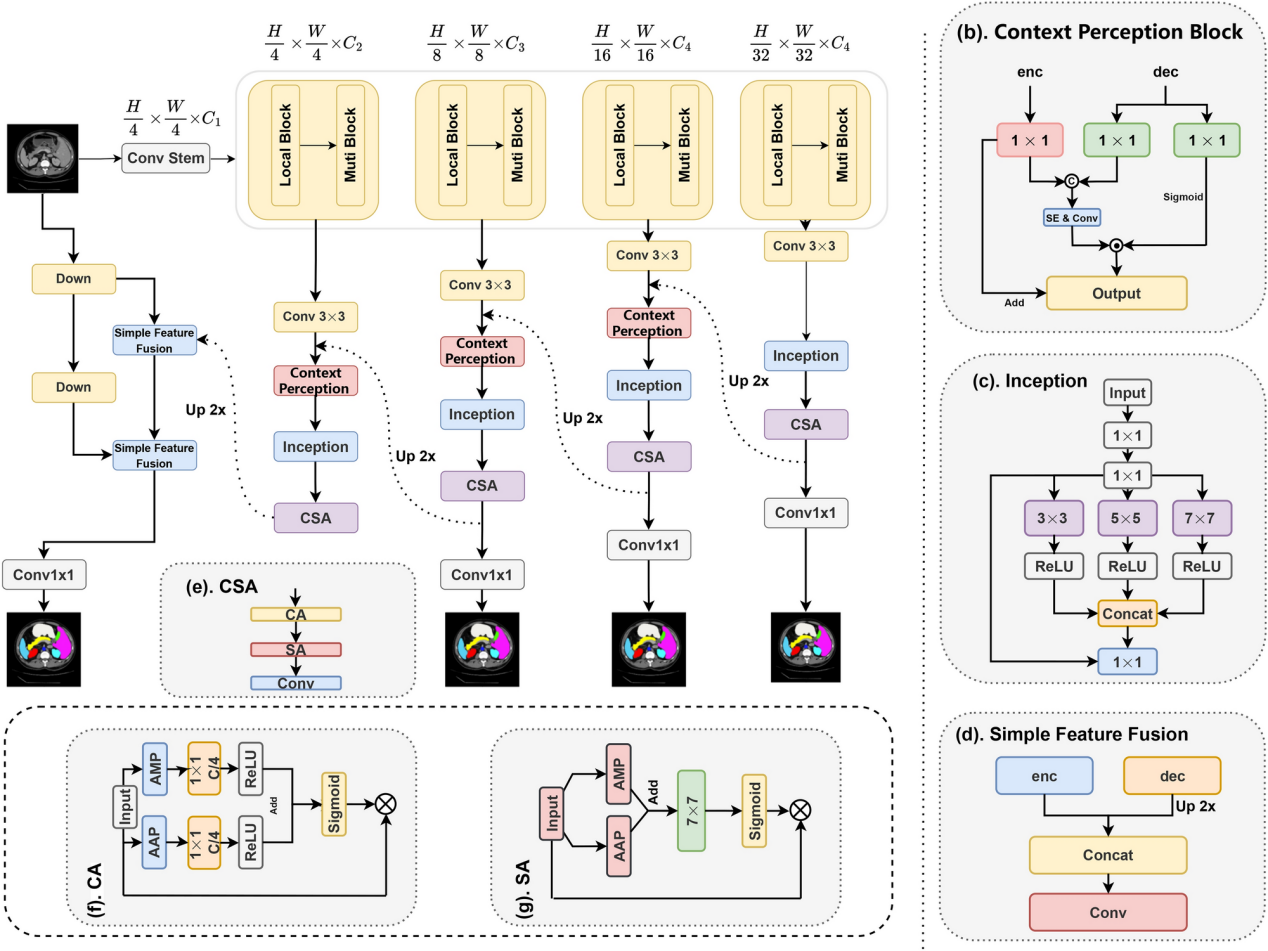
(**a**) The architecture of ScaleFormer, which is composed of encoder, decoder. Encoder based on patch embedding, multi block. (**b**) Description of efficient feature fusion block. (**c**) Description of inception block. (**d**) Description of simple feature fusion block. (**e**) Description of CSA block. (**f**) Description of CA block. (**g**) Description of SA block.

**Fig. 3:**
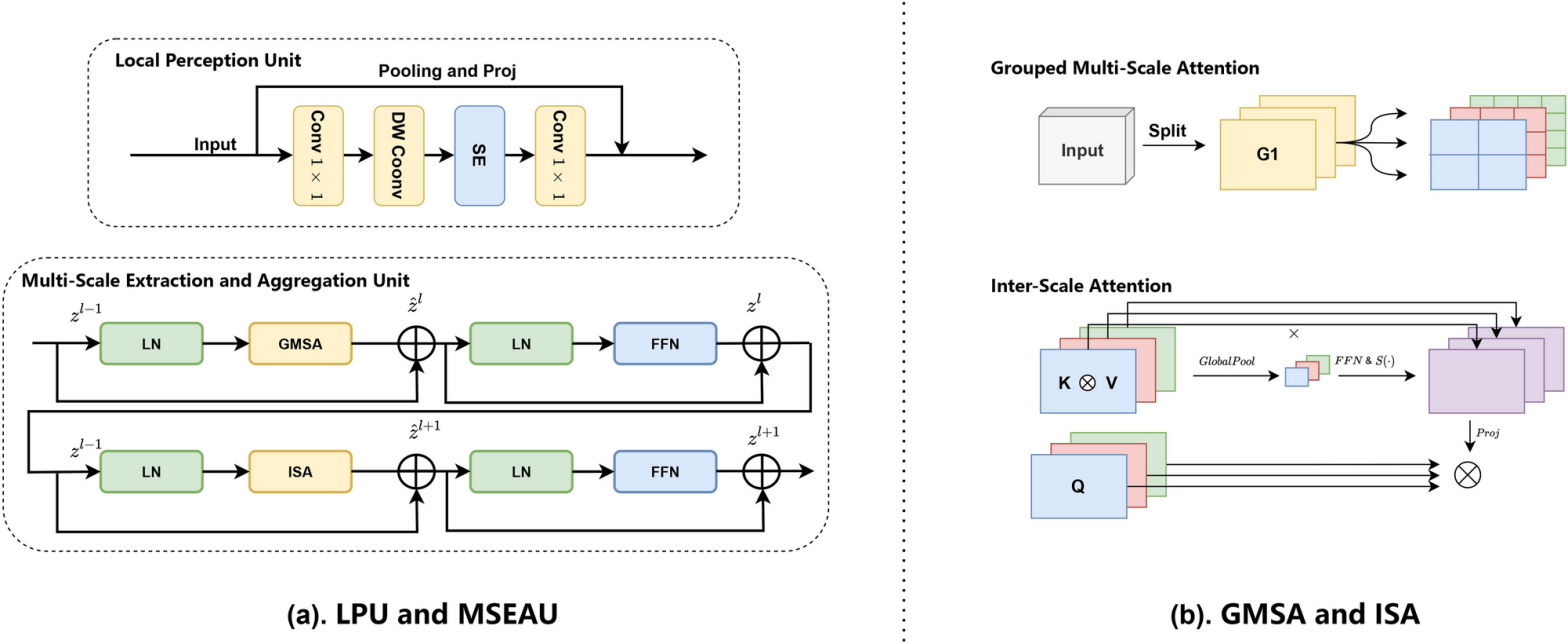
(**a**) Description of the LPU and MSEAU. (**b**) Description of the grouped multi-scale attention and inter-scale attention. $S (\cdot )$ represents the sigmoid activation function.

## Proposed method

The proposed ScaleFormer is a traditional architecture that consists of an encoder and a decoder, as illustrated in Fig. 2. We design the Local Perception Unit (LPU) based on convolution and constructed the Multi-Scale Extraction and Aggregation Unit (MSEAU) using Grouped Multi-Scale Attention (GMSA) and Inter-Scale Attention (ISA), as shown in Fig. 3. Our hierarchical encoder can be constructed based on the above modules. Our decoder is crafted to restore prediction results to the same resolution as the input. The architecture is composed entirely of efficient convolutional blocks and includes several key components: an Context Perception Block to integrate encoder and decoder features, an Inception structure for extracting rich multi-scale features, a CSA structure to emphasize both spatial and channel information, and a simple feature fusion structure designed to enhance fine details in high-resolution features. The next section will provide a more detailed description of our method.

### Encoder

In the $i$th stage of the proposed hierarchical encoder, there is one Local Perception Unit(LPU) and $L_i$ Multi-Scale Extraction and Aggregation Unit(MSEAU). Each MSEAU consists of a pair of consecutive Grouped Multi-Scale Attention (GMSA) and Feed-Forward Network (FFN) as well as Inter-Scale Attention (ISA) and FFN.

Local perception unit: Rotation and shifting are widely used data augmentation techniques in visual tasks, and these operations should not affect the model’s final output. In other words, our goal is to attain invariance to translation in these tasks^22^. However, self-attention inherently lacks this characteristic. Furthermore, Vits tend to overlook local relationships within patches^23^ and structural information^24^. To address these limitations, we introduce a LPU into each encoder to incorporate these properties and supplement local information. The LPU is defined as follows:

1 $$\begin{aligned} \operatorname {LPU}(X) = \operatorname {Skip}(X) + \operatorname {Proj} \big ( \operatorname {SE} \big ( \operatorname {DWConv} (\operatorname {Conv} ( \operatorname {Norm}(X))) \big ) \big ) \end{aligned}$$

where Norm is BatchNorm^25^, Conv is the expansion Conv $1 \times 1$ followed by BatchNorm and GELU^26^ activation, a typical choice for Transformer-based models. DWConv is the Depthwise Conv $3 \times 3$ followed by BatchNorm and GELU. $\text {Skip}(\cdot )$ is either a pooling layer or an identity layer, depending on whether downsampling is performed. SE is the Squeeze-Excitation layer^27^, while Proj is the shrink Conv $1 \times 1$ to down project the number of channels.

Multi-scale extraction and aggregation unit: The MSEAU is built using the GMSA and ISA modules. In the GMSA module, the input $X \in {\mathbb {R}}^{h \times w \times c}$ is first divided into $G$ groups along the channels. In each group, every input $x_i$ is initially projected into query (Q), key (K), and value (V) tensors:

2 $$\begin{aligned}&x \rightarrow \{x_1, x_2, \ldots , x_g\} \end{aligned}$$

3 $$\begin{aligned}&Q_i, K_i, V_i = x_i W^Q_i, x_i W^K_i, x_i W^V_i \end{aligned}$$

here the $W^Q_i$, $W^K_i$, $W^V_i$are the parameters of the linear projection in the $i$-th group. As shown in Fig. 3b, different groups of $\{Q, K, V\}$ correspond to patches of varying sizes. Specifically, for $group_i$, the patch size is determined by $s_i$ and computed as $(H_i/s_i, W_i/s_i)$, resulting in an effective sequence length of $N_i = s_i^2$. By assigning different $s_i$ values to each group, we introduce multiple receptive field sizes within a single attention layer, enhancing multi-scale feature extraction. Unlike conventional self-attention, which uses a fixed patch size with a computational complexity of $O(N^2d)$ (where *N* is the token count), our approach determines patch dimensions via $s_i$. This leads to a per-group complexity of $O(N_i^2d) = O(s_i^4d)$, and summing over all *G* groups gives a total complexity of $O\Bigl (\sum _{i=1}^{G} s_i^4d_i\Bigr )$. A smaller $s_i$ captures larger patches, while a larger $s_i$ retains finer details at a higher computational cost. By integrating multiple scales within a single self-attention layer and varying $s_i$ across attention heads, our method enriches feature representation across different granularities.

The ISA module is illustrated in Fig. 3b. Given an input $x$, we first map it to $\{Q, K, V\}$ following a similar procedure as in previous self-attention operations. The input is then divided into multiple groups, denoted as $Q_i, K_i, V_i$, and a Softmax operation is applied to $Q_i$ and $K_i$, following the channel-aware attention mechanism^28^. The attention weights for each group are computed through operations on $K$ and $V$. To capture global contextual relationships across different groups, a global pooling operation is applied to aggregate attention information, generating a single representative value for each group’s attention correlation. These values are concatenated and processed by a feed-forward network (FFN) to produce a scaling factor. The computed scaling factor is then applied to each attention map via point-wise multiplication, followed by concatenation of the refined attention maps. Finally, the aggregated attention information is integrated into each $Q_i$, enabling cross-group information exchange. This process can be described as follows:

4 $$\begin{aligned}&X \rightarrow \{Q, K, V\} \end{aligned}$$

5 $$\begin{aligned}&Q \rightarrow \{ \text {Softmax}(q_1), \text {Softmax}(q_2), \ldots , \text {Softmax}(q_g) \} \end{aligned}$$

6 $$\begin{aligned}&K \rightarrow \{ \text {Softmax}(k_1), \text {Softmax}(k_2), \ldots , \text {Softmax}(k_g) \} \end{aligned}$$

7 $$\begin{aligned}&CA = K V^T \end{aligned}$$

8 $$\begin{aligned}&A = \text {Proj}\left( CA \times \text {Sigmoid}\left( \text {FFN}\left( \text {Global Pool}(CA)\right) \right) \right) \end{aligned}$$

9 $$\begin{aligned}&\text {F} = \text {Concatenate}\left\{ A^T q_1, A^T q_2, \ldots , A^T q_g \right\} \end{aligned}$$

### Decoder

We have developed a feature-rich decoder to restore image information and generate the final segmentation mask, as shown in Fig. 2. For each decoder stage output $d_i$, we integrate it with feature maps from the encoder at the same resolution using the Context Perception Block (CPB) module to recover image details. We then use an Inception module, composed of multiple convolutional layers at different scales, to extract rich multi-scale information. Prior to generating the final segmentation mask, channel attention and spatial attention mechanisms are applied to emphasize spatial and channel features. The CPB module takes outputs from both the encoder and the decoder, applies a Squeeze-and-Excitation (SE) layer to learn channel features, and then uses convolution to fuse these channel features into a combined result $x'$. We generate a gating unit from the decoder output using a sigmoid function and multiply it with $x'$ to dynamically adjust the spatial attention weights in an adaptive manner. Finally, the result is combined with the original encoder output to produce the final output, which helps stabilize gradients and mitigate the issue of gradient vanishing. It is important to note that we do not use the CPB module at the last layer of the encoder stage, as the decoder output is not yet available. On the other hand, directly using a 4x upsampling operator can lead to a significant loss of shallow features. Therefore, we downsample the input image by cascading two blocks to obtain low-level features with resolutions of *H* $\times$ *W* and $\frac{H}{2}$ $\times$ $\frac{W}{2}$, respectively. Each block consists of a $3\times 3$ Conv layer, a BatchNorm layer, and a ReLU layer in sequence. All these output features will be utilized to obtain the final mask prediction through skip connections.

### Loss function

We used a combination of cross entropy (CE) loss and Dice loss to train ScaleFormer. The total loss ${\mathscr {L}}$ between the prediction ${\hat{y}}$ and the target *y* is formulated as:

10 $$\begin{aligned} {\mathscr {L}} = 0.3 \cdot \text {CELoss}({\hat{y}}, y) + 0.7 \cdot \text {DiceLoss}({\hat{y}}, y) \end{aligned}$$

where $\text {CELoss}({\hat{y}}, y)$ is the cross-entropy loss and $\text {DiceLoss}({\hat{y}}, y)$ is the Dice loss, defined as:

11 $$\begin{aligned} \text {DiceLoss}({\hat{y}}, y) = 1 - \frac{2 \cdot |{\hat{y}} \cap y|}{|{\hat{y}}| + |y|} \end{aligned}$$

In this equation, ${\hat{y}}$ represents the predicted segmentation mask and *y* represents the ground truth. The parameter ${\mathscr {L}}$ is a weighted sum of the two loss functions, where the cross-entropy loss contributes 30% and the Dice loss contributes 70%. In our training process, we also employed a multi-scale training strategy, and the final loss function ${\mathscr {L}}_{\text {total}}$ can be expressed as follows:

12 $$\begin{aligned} {\mathscr {L}}_{\text {total}} = \alpha {\mathscr {L}}_{\text {1}} + \beta {\mathscr {L}}_{\text {2}} + \gamma {\mathscr {L}}_{\text {3}} + \delta {\mathscr {L}}_{\text {4}} \end{aligned}$$

The loss components are defined as follows: ${\mathscr {L}}_{\text {1}}$ is obtained from the final branch, ${\mathscr {L}}_{\text {2}}$ is derived from the $\frac{H}{8} \times \frac{W}{8}$ branch, ${\mathscr {L}}_{\text {3}}$ originates from the $\frac{H}{16} \times \frac{W}{16}$ branch, and ${\mathscr {L}}_{\text {4}}$ comes from the $\frac{H}{32} \times \frac{W}{32}$ branch. The hyperparameters $\alpha$, $\beta$, $\gamma$, and $\delta$ are empirically set to 0.7, 0.1, 0.1, and 0.1, respectively.

## Experiments and results

Our proposed method is implemented in an end-to-end manner using the PyTorch library and is trained on a single RTX 3090 GPU.

### Datasets

We evaluated our model on three publicly available datasets: **Synapse**, **ACDC**, and **ISIC2018**.

**Synapse**: Synapse contains 30 abdominal CT scans (3,779 axial slices) covering multiple organs (e.g., aorta, liver, pancreas). Following TransUnet^12^, we used 18 scans (2,212 slices) for training and 12 (1567 slices) for validation.

**ACDC**: ACDC consists of 100 cardiac MRI scans with three segmentation targets: right ventricle (RV), left ventricle (LV), and myocardium (Myo). We followed TransUnet’s setup, using 70 cases (1,930 slices) for training, 10 for validation, and 20 for testing.

**ISIC2018**: ISIC2018 includes 2694 dermoscopy images with segmentation masks. Since prior studies used varying data splits, we adopted the EGE-UNet^29^ setup: 1886 images for training and 808 for testing, ensuring fair comparison.

### Implementation details

For all the datasets, we used a batch size of 12. We followed the baseline design and used different optimization methods for different datasets. For the ACDC dataset, we utilized the Adam optimizer with a learning rate of 0.0001 and weight decay of 0.0001. Meanwhile, for the Synapse dataset, we trained the model using stochastic gradient descent (SGD) with a base learning rate of 0.05, a momentum of 0.9, and a weight decay of 0.0001. For ISIC2018 dataset , all images were resized to 256 *times* 256, and we utilized the Adam optimizer with a learning rate of 0.0001 and weight decay of 0.0001. These hyperparameters have been commonly used in the literature^12^.

### Evaluation metrics

Following existing methods, we used DICE, 95% Hausdorff Distance (95HD) as the evaluation metrics in our experiments on Synapse Multi-organ dataset. For the ACDC dataset, since the HD95 metric is not effective in distinguishing model performance, we followed the TransUnet approach, which only utilized DICE scores for the ACDC dataset. As for the ISIC2018 dataset, existing methods^29,30^ typically use mean Intersection over Union (mIoU), Dice coefficient, Accuracy (Acc), Sensitivity (Sen), and Specificity (Spe) as evaluation metrics. We followed this approach without making any changes.

**Table 1:** Comparison results of the proposed method on the Synapse dataset $\uparrow$ denotes higher the better, $\downarrow$ denotes lower the better. The best result is in bold, and the second best is in italics. We adopt a 2D image slicing approach rather than 3D, so only 2D models are listed.

Methods	DSC $\uparrow$	HD $\downarrow$	Aorta	Gallbladder	Kidney(L)	Kidney(R)	Liver	Pancreas	Spleen	Stomach
UNet^5^	76.85	39.70	**89**.**07**	69.72	77.77	68.60	93.43	53.98	86.67	75.58
R50-ViT^12^	71.29	32.87	73.73	55.13	75.80	72.20	91.51	45.99	81.99	73.95
TransUNet^12^	77.48	31.69	87.23	63.13	81.87	77.02	94.08	55.86	85.08	75.62
MT-Unet^31^	78.59	26.59	87.92	64.99	81.47	77.29	93.06	59.46	87.75	76.81
Swin Unet^13^	79.13	21.55	85.47	66.53	83.28	79.61	94.29	56.58	90.66	76.60
LeVit-Unet^32^	78.53	16.84	78.53	62.23	84.61	80.25	93.11	59.07	88.86	72.76
MISSFormer^33^	81.96	18.20	86.99	68.65	85.21	82.00	94.41	65.67	91.92	80.81
ScaleFormer (Huang)^34^	82.86	16.81	88.73	**74**.**97**	86.36	83.31	95.12	64.85	89.40	80.14
HiFormer^35^	80.69	19.14	87.03	68.61	84.23	78.37	94.07	60.77	90.44	82.03
DAE-Former^36^	82.43	17.46	88.96	72.30	86.08	80.88	94.98	65.12	91.94	79.19
PVT-CASCADE^37^	81.06	20.23	83.01	70.59	82.23	80.37	94.08	64.43	90.10	*83.69*
TransCASCADE^37^	82.68	17.34	86.63	68.48	*87.66*	*84.56*	94.43	65.33	90.79	83.52
MAXFormer^38^	83.66	15.89	87.72	73.53	87.92	**84**.**67**	95.00	66.55	92.46	81.44
LaplacianFormer^39^	81.90	18.66	86.55	71.19	84.23	80.52	94.90	64.75	91.91	81.14
PVT-GCASCADE^40^	83.28	15.83	86.50	71.71	87.07	83.77	95.31	66.72	90.84	83.58
PVT-EMCAD^41^	83.63	15.68	88.14	68.87	**88**.**08**	84.10	95.26	**68**.**51**	92.17	**83**.**92**
ScaleFormer(Ours)	**84**.**00**	**12**.**30**	*88.98*	*74.92*	87.33	84.51	**95**.**48**	*66.82*	**92**.**88**	81.04

**Fig. 4:**
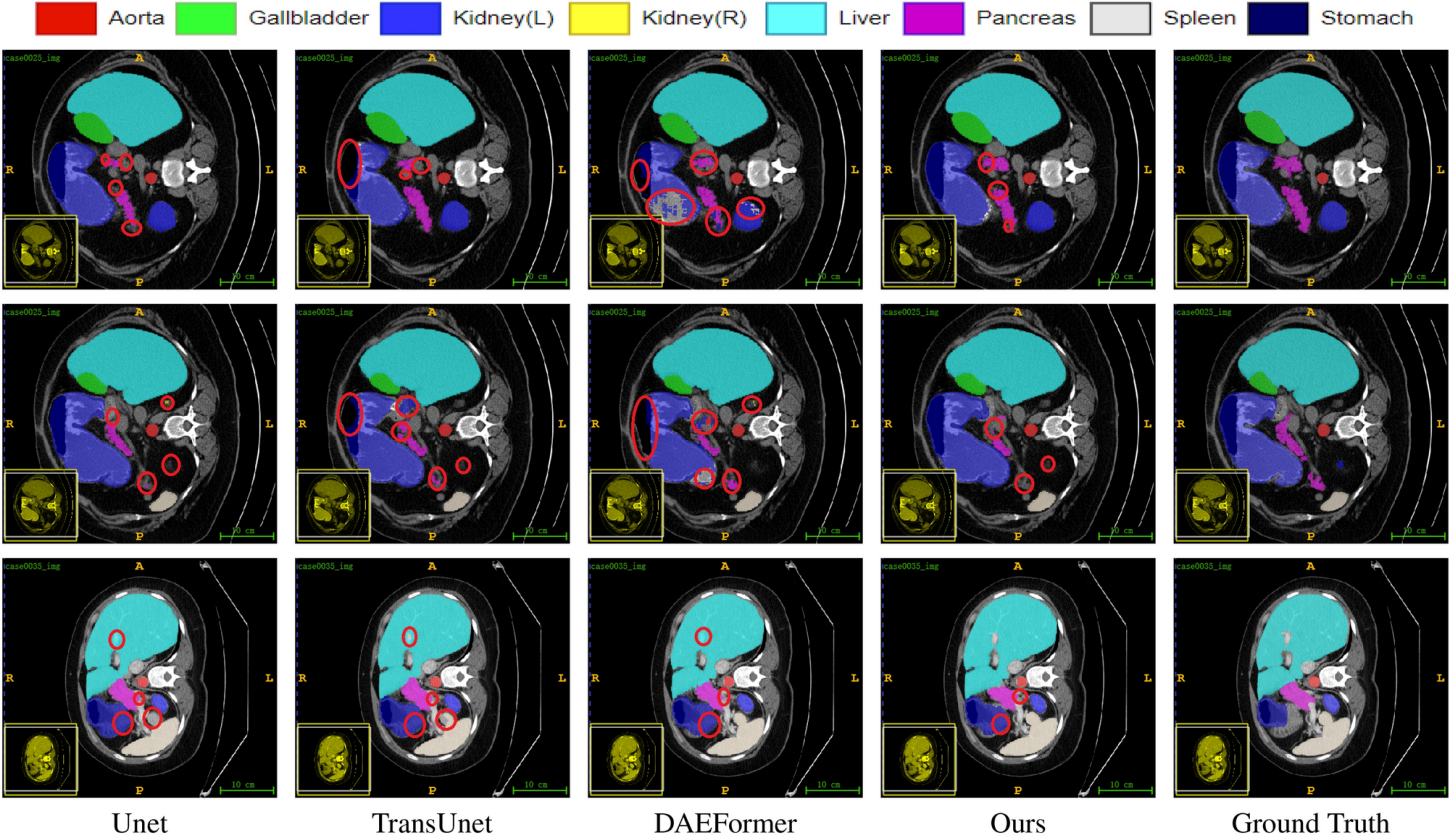
Comparative segmentation results on the Synapse dataset. The red mark circles defect parts of the predictions.

### Results

The experimental results on the Synapse dataset are presented in Table 1 and Fig. 4. Our method outperforms several existing advanced models on this dataset, particularly in terms of the Dice coefficient and HD95 metrics, achieving 84.00 and 12.30, respectively, surpassing most comparison methods. The improvement in Dice scores reflects the significant enhancement in overall segmentation accuracy, especially in capturing the overall shape and structure of organs. Meanwhile, the reduction in HD95 indicates substantial progress in precisely delineating organ boundaries. This demonstrates that our model has a notable advantage in extracting both overall features and boundary details of organs. Figure 4 illustrates the segmentation results of our method across multiple organs. Most of the segmentation results are smooth and align well with the actual anatomical structures, highlighting the potential of our model in the domain of organ segmentation. Specifically, our method exhibits outstanding performance in large organ segmentation tasks, such as the aorta, liver, and spleen. Furthermore, it also surpasses most previous results in the stomach segmentation task. In addition, for small organ segmentation tasks, our method performs remarkably well in the segmentation of the kidneys and gallbladder, achieving the satisfactory results, particularly in gallbladder segmentation. This performance improvement is primarily attributed to our proposed GMSA and ISA methods. The GMSA module enhances organ feature representation across different scales through a grouped multi-scale attention mechanism. Meanwhile, the ISA module expands the learning scope of multi-scale features by enabling interaction among grouped features, thereby improving the model’s ability to learn features and segmentation precision across various types of organs.

However, although our method demonstrates superior performance on most metrics, there remains room for improvement in certain local regions. Specifically, in large-organ segmentation tasks, such as the segmentation of the aorta and stomach, our method fails to outperform other existing methods completely, particularly underperforming in stomach segmentation. This could be attributed to the complex shapes of these organs and their similar textures to surrounding tissues, which limits the model’s ability to accurately delineate their boundaries during the decoding stage due to constraints in detail recovery. On the other hand, in the pancreas segmentation task, although our model achieves commendable performance in terms of the Dice coefficient, there is still a gap compared to some state-of-the-art methods, as validated in Fig. 4. This indicates that our model has room for optimization when addressing segmentation tasks involving low contrast and blurred boundaries, especially in enhancing the model’s sensitivity to low-contrast regions and its ability to refine boundaries.

**Table 2:** Comparison results of the proposed method on the ACDC dataset. $\uparrow$ denotes higher the better, $\downarrow$ denotes lower the better. The best result is in bold, and the second best is in italics.

Methods	DSC $\uparrow$	RV	Myo	LV
R50-UNet^12^	87.60	84.62	84.52	93.68
R50-AttnUNet^12^	86.90	83.27	84.33	93.53
TransUnet^12^	89.71	86.67	87.27	95.18
Swin Unet^13^	90.00	88.55	85.62	*95.83*
ScaleFormer(Huang)^34^	90.17	87.33	88.16	95.04
MT-UNet^31^	90.43	86.64	89.04	95.62
MEW-UNet^42^	91.00	88.82	88.61	95.56
PVT-CASCADE^37^	91.46	*89.97*	88.90	**95**.**90**
TransCASCADE^37^	*91.63*	89.14	**90**.**25**	95.50
SSTrans-Net^43^	90.31	89.02	87.51	94.40
ScaleFormer(ours)	**91**.**92**	**90**.**08**	*89.90*	95.78

**Fig. 5:**
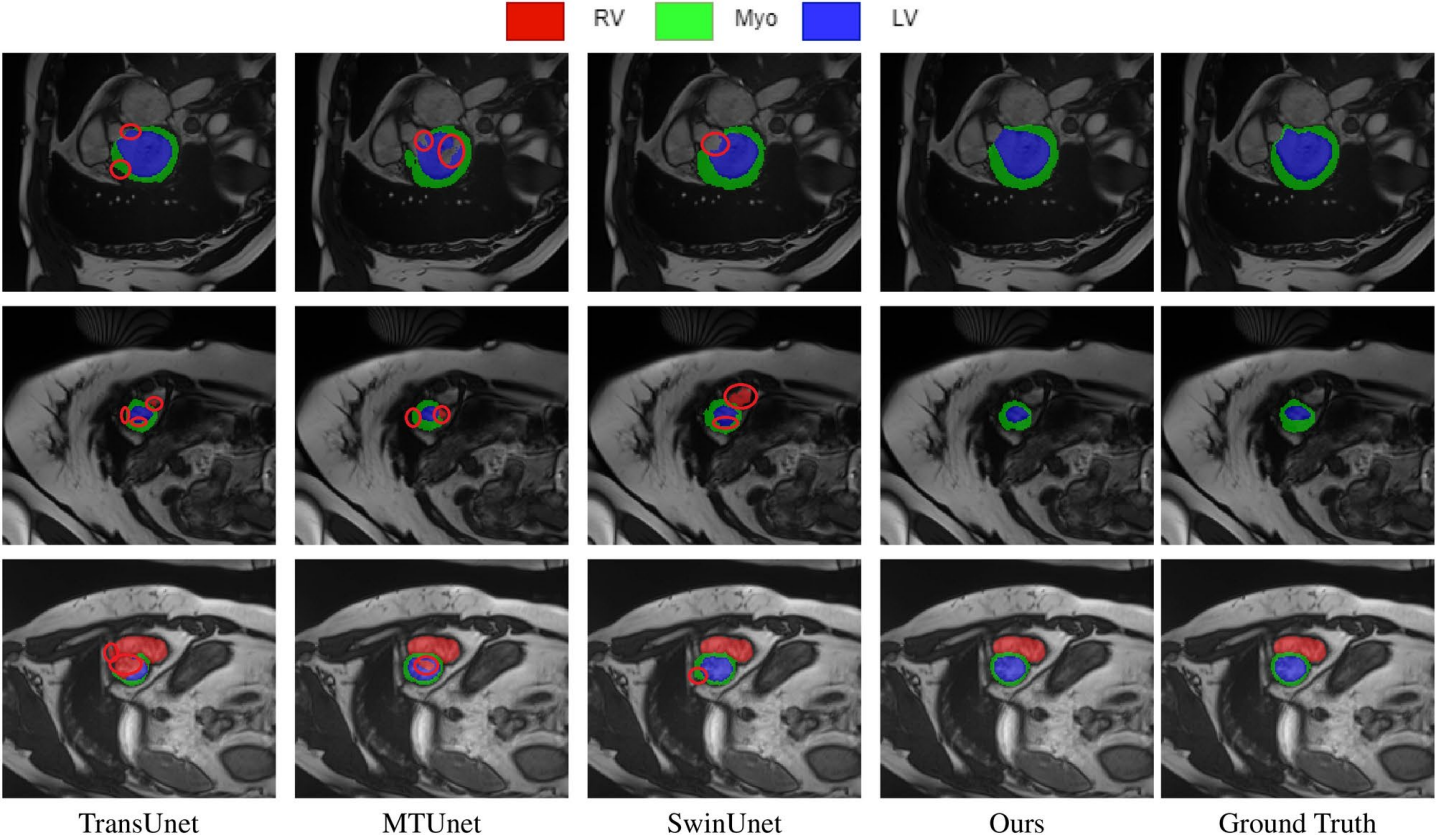
Comparative segmentation results on the ACDC dataset. The red mark circles defect parts of the predictions.

**Fig. 6:**
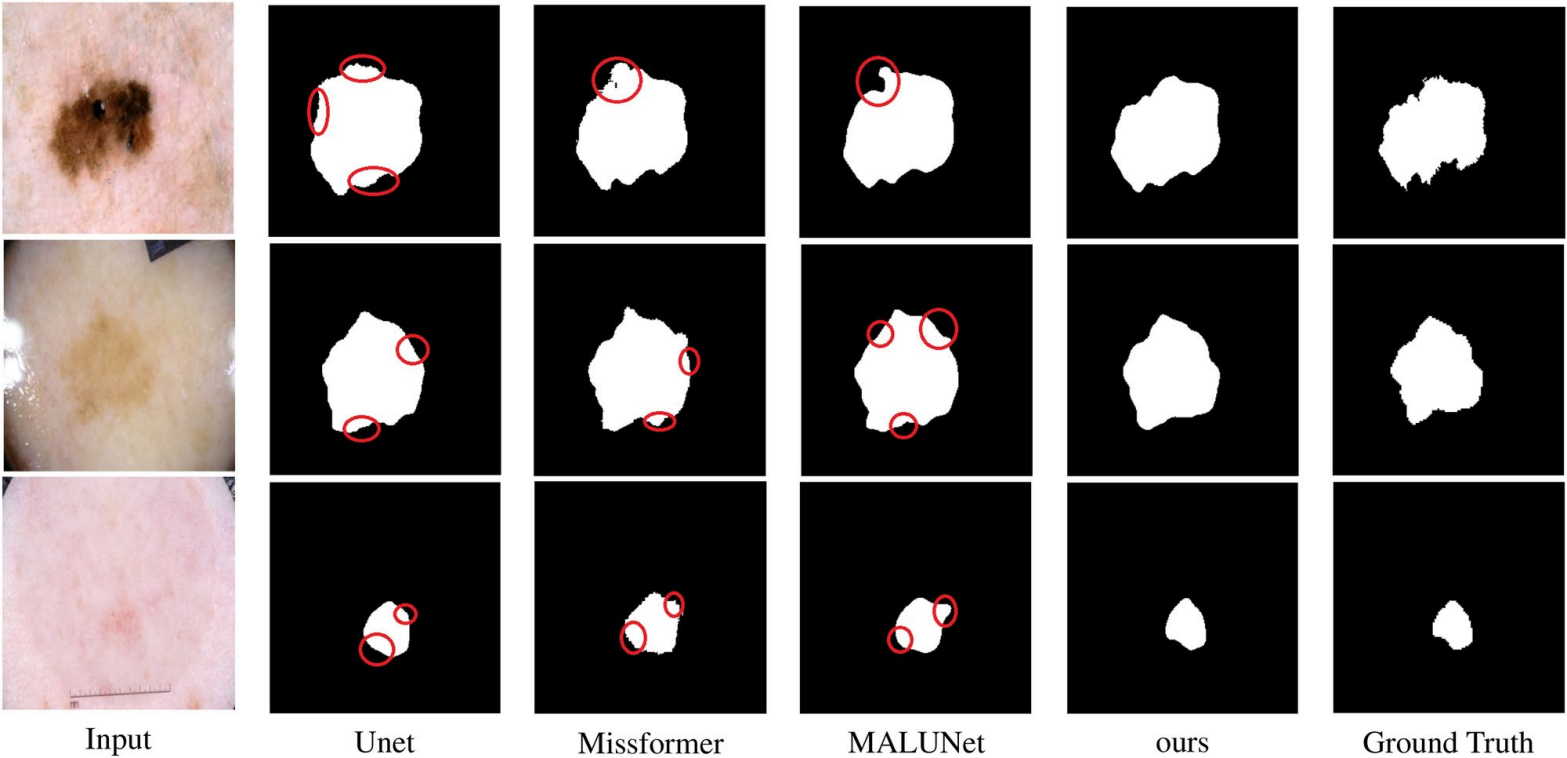
Comparative segmentation results on the ISIC2018 dataset. The red mark circles defect parts of the predictions.

Tables 2 and 3 present the experimental results of our method on the ACDC and ISIC2018 datasets, respectively. Additionally, Figs. 5 and  6 provide the corresponding visual results for these datasets, highlighting the performance of our method. The results demonstrate that our approach excels across multiple evaluation metrics. On the ACDC dataset, as shown in Table 2, our method achieved a Dice coefficient of 91.92, surpassing all compared methods. Notably, in the segmentation tasks for the left ventricle (LV) and myocardium (Myo), we achieved consistent performance, with the Dice coefficient for the left ventricle (LV) reaching 95.78, delivering the best segmentation results. However, for the right ventricle (RV) segmentation task, although our Dice coefficient of 90.08 ranks as the second-best, there remains a gap compared to the most advanced model. We attribute this primarily to the fuzzy boundaries of the right ventricle and the superior performance of SSTrans-Net in handling small-structure segmentation tasks. From the experimental results, it is evident that while our method has achieved remarkable overall segmentation accuracy, it still lags behind the state-of-the-art models in segmenting smaller structures.

**Table 3:** Comparison results of the proposed method on the ISIC2018 dataset (mean ± std). $\uparrow$ denotes higher the better, $\downarrow$ denotes lower the better. The best result is in bold, and the second best is in italics.

Methods	mIoU$\uparrow$	DSC$\uparrow$	Acc$\uparrow$	Spe$\uparrow$	Sen$\uparrow$
UNet^5^	0.7757±0.1705	0.8737±0.1348	0.9392±0.0910	0.9634±0.0980	0.8638±0.1612
Attention-UNet^44^	0.7766±0.1703	0.8743±0.1353	0.9395±0.0869	0.9638±0.0865	0.8639±0.1616
TransFuse^45^	0.7791±0.1647	0.8758±0.1327	0.9392±0.0780	0.9574±0.0870	**0.9128** ± **0.1558**
TransUNet^12^	0.7922±0.1602	0.8840±0.1275	0.9439±0.0781	0.9647±0.0918	0.8791±0.1586
Swin-UNet^13^	0.7824±0.1701	0.8779±0.1359	0.9408±0.0790	0.9626±0.0878	0.8731±0.1664
DCSAUNet^46^	0.7934±0.1709	0.8848±0.1386	0.9440±0.0873	0.9634±0.1038	0.8836±0.1609
MALUNet^47^	0.8025±0.1582	0.8904±0.1255	0.9462±0.0782	0.9619±0.0867	0.8974±0.1460
FocalUNETR^48^	0.7834±0.1799	0.8785±0.1443	0.9426±0.0844	0.9614±0.0619	0.8528±0.1696
DconnNet^30^	*0.8094 ±0.1554*	*0.8947 ± 0.1241*	*0.9484 ± 0.0720*	0.9641±0.0979	0.8997±0.1429
EGE-UNet^29^	0.8000±0.1642	0.8888±0.1317	0.9461±0.0772	**0.9658±0.0976**	0.8849±0.1536
I2U-Net^49^	0.8031±0.1570	0.8908±0.1220	0.9469±0.0750	*0.9655 ± 0.0838*	0.8890±0.1443
**ScaleFormer (ours)**	**0.8146**±**0.1588**	**0.8978** ± **0.1249**	**0.9498** ± **0.0712**	0.9641±0.1008	*0.9056±0.1418*

Table 3 and Fig. 6 illustrate the performance of our method on the ISIC2018 dataset. The figure visually demonstrates the accuracy of our method in restoring the boundaries and shape details of skin lesions. Although our method achieved improvements over previous approaches with mIoU, Dice coefficient, and accuracy (Acc) reaching 81.46, 89.78, and 94.98, respectively. However, certain methods However, such as EGE-UNet and TransFuse, yield superior results in specificity (Spe) and sensitivity (Sen). Notably, TransFuse achieved a high sensitivity score of 91.28, significantly surpassing our method. These results suggest that while our model performs well across most evaluation metrics, there is still room for improvement in segmentation tasks involving blurred boundaries.

Overall, our method demonstrated satisfactory performance on both the ACDC and ISIC2018 datasets, showing particular strengths in large-organ localization and overall boundary segmentation, which highlights the model’s strong generalization ability. These experimental results indicate that our model effectively enhances segmentation accuracy in complex medical image segmentation tasks and performs well across multiple metrics, showcasing its potential in the field of medical image segmentation.

### Statistical significance test

**Table 4:** Paired t-test results and confidence intervals comparing ScaleFormer with other models.

Methods	T-statistic	P-value	Significant	Confidence
Unet	4.5620	< 0.01	Yes	(0.8642, 0.8828)
TransUnet	2.4532	0.0144	Yes	(0.8724, 0.8900)
SwinUnet	5.3701	< 0.01	Yes	(0.8598, 0.8786)
DCSAUNet	3.1733	< 0.01	Yes	(0.8694, 0.8885)
MALUNet	2.1521	0.0317	Yes	(0.8731, 0.8904)
FocalUNETR	4.9210	< 0.01	Yes	(0.8604, 0.8803)
EGE-UNet	2.0269	0.0430	Yes	(0.8730, 0.8912)
I2UNet	2.0840	0.0375	Yes	(0.8724, 0.8904)
ScaleFormer(Ours)	–	–	–	(0.8799, 0.8971)

To further validate the effectiveness of our proposed method, we conducted a paired t-test between ScaleFormer and other models on the ISIC2018 dataset. The results are summarized in Table 4. We employed paired sample t-tests to assess performance differences, analyzing them using the t-statistic and p-value. The t-statistic quantifies the difference in mean performance between two models relative to the standard error, with a higher absolute value indicating a more pronounced difference. The p-value measures the likelihood that the observed differences arise purely by chance, with p < 0.05 signifying statistical significance. Additionally, we calculated 95% confidence intervals to provide a reliable measure of the observed differences, ensuring the robustness of our results. The comparative models included CNN-based architectures (UNet, Attention-UNet), Transformer-based methods (TransUNet, Swin-UNet), and more recently proposed cutting-edge approaches with significant research value (MALUNet, EGE-UNet, I2UNet). From the results, we observe that ScaleFormer consistently outperforms existing models with statistically significant improvements. Specifically, the p-values for all comparisons are below 0.05, indicating that the performance differences are unlikely due to random variations. Notably, models such as UNet, Swin-UNet, and FocalUNETR exhibit particularly large t-values (e.g., 4.5620, 5.3701, and 4.9210, respectively), with p-values below 0.01, confirming highly significant differences. Furthermore, the confidence intervals demonstrate that ScaleFormer achieves superior Dice scores across all comparisons, further reinforcing the robustness of our method. These findings validate that the observed performance gains of ScaleFormer are not only meaningful but also statistically significant, ensuring the reliability of our proposed approach in medical image segmentation tasks.

## Ablation studies

We conducted a series of ablation experiments to investigate the influence of our proposed approach on the overall model architecture. The results of these experiments are presented in Table 5.

**Table 5:** Ablation Study on the modules with Synapse datasets. The best result is in bold, and the second best is in italics.

Components	DSC $\uparrow$	HD $\downarrow$
W/O Multi-Scale	82.41	17.41
W/O ISA module	82.70	17.26
W/O LPU	82.80	*15.75*
W/O CPB	83.11	16.04
W/O Inception	*83.30*	16.20
Ours	**84**.**00**	**12**.**30**

We compared the performance impact of using a single-scale partitioning instead of multi-scale Attention. The results showed a 1.59% decrease in DSC and a 5.11% increase in HD95, indicating that single-scale partitioning reduces overall segmentation quality and produces less smooth boundaries. This is mainly because the Synapse dataset contains organs with varying shapes and sizes, and the multi-scale approach better captures these variations. We then compared the model’s performance without the ISA mechanism, which resulted in a 1.3% decrease in DSC and a 4.96% increase in HD95. This performance drop is primarily due to the network’s inability to transmit and fuse multi-scale information across different heads without the GMSA structure. Next, we evaluated the impact of using versus not using the LPU. The results clearly showed that models incorporating the LPU performed better. This improvement can be attributed to the convolution-based LPU, which introduces translation invariance, accelerates the convergence of the self-attention module, and enhances the model’s ability to capture local spatial information.

We also conducted an ablation study on the main structure of our Decoder module. First, we compared the model’s performance without the CPB module, replacing it with a simple concatenation and convolution block. The results indicate that the model with the CPB module outperformed the alternative, as the CPB integrates channel and spatial attention mechanisms, enabling more selective focus on feature information compared to traditional concatenation methods. Additionally, we tested the impact of the Inception module. The results demonstrated that the network incorporating the Inception module performed better. This improvement can be attributed to the fact that the upsampling process often leads to the loss of multi-scale information, and the Inception module helps mitigate this issue by effectively capturing features at multiple scales.

**Table 6:** Ablation study on scale configurations. Each row represents a different multi-scale grouping strategy across four stages. [s1, s2, s3, s4] denotes the scaling factors, where each group is assigned a patch size of $(H_i/s_i, W_i/s_i)$. The best result is in bold, and the second best is in italics.

config	Stage 1	Stage 2	Stage 3	Stage 4	DSC $\uparrow$	HD $\downarrow$
	[7, 7, 7, 7]	[4, 4, 4, 4]	[2, 2]	[2, 2]	82.41	17.41
	[1, 2, 4, 7]	[1, 2, 4, 7]	[1, 7]	[1, 7]	**84**.**00**	**12**.**30**
	[7, 7, 7, 7]	[1, 2, 4, 7]	[1, 7]	[1, 7]	83.28	15.76
	[7, 7, 7, 7]	[4, 4, 4, 4]	[1, 7]	[1, 7]	82.85	14.30
	[7, 7, 7, 7]	[4, 4, 4, 4]	[1, 2, 7]	[1, 7]	82.76	14.27
	[2, 4, 7, 14]	[2, 4, 7, 14]	[1, 7]	[1, 7]	*83.48*	*12.88*

Additionally, we conducted a series of ablation experiments on the Synapse dataset to analyze the impact of different scale selection configurations on model performance. As shown in Table 6, using a single-scale grouping strategy (first row) leads to suboptimal results (Dice: 82.41, HD95: 17.41), suggesting limited adaptability to varying anatomical structures. In contrast, incorporating multi-scale grouping significantly improves segmentation performance, achieving a Dice score of 84.00 and an HD95 of 12.30. This highlights the advantage of capturing multi-scale contextual features in medical images. Further analysis shows that multi-scale grouping in shallow layers is particularly beneficial, as high-resolution feature maps preserve fine details such as edges and textures. Conversely, in deeper layers, where spatial resolution is already low (e.g., $7\times 7$ in Stage 4), different scale configurations have a less pronounced effect. Notably, we experimented with larger scaling factors that produce smaller patches per stage. However, this setup led to reduced performance. We hypothesize that excessively small patch sizes hinder the establishment of long-range dependencies, ultimately limiting the model’s capacity to capture global contextual information.

## Discussion

While our proposed method demonstrates competitive segmentation performance, some limitations remain.

In “Encoder”, we analyzed the computational cost of the proposed Group Multi-Scale Attention (GMSA) module. While the cost of GMSA can be adjusted via *s*, the overall computational overhead remains relatively high. As shown in Table 6, our method achieves a Dice score of 84.00 and an HD95 of 12.30, outperforming previous approaches in segmentation accuracy while maintaining a lower computational cost than competitive multi-scale models such as MAXFormer. However, the total number of parameters (69.00M) and FLOPs (40.72G) are still higher than those of more lightweight architectures like PVT-CASCADE (35.27M, 8.20G). This increase in complexity primarily stems from the additional components integrated into our framework to enhance feature extraction and spatial representation. To further illustrate this, we replaced our decoder with the CASCADE decoder from PVT-CASCADE, forming ScaleFormer-CASCADE. The results indicate that ScaleFormer-CASCADE maintains superior Dice and HD95 scores while achieving a computational cost comparable to PVT-CASCADE. This suggests that decoder optimization plays a crucial role in balancing performance and efficiency. Future work could focus on designing more efficient decoder mechanisms or employing model pruning strategies to reduce computational complexity while maintaining performance (Table 7).

**Table 7:** Comparison of computational costs between ScaleFormer and competing methods. The best result is in bold, and the second best is in italics.

Methods	Params	Flops	DSC $\uparrow$	HD $\downarrow$
U-Net	37.67M	52.09	76.85	39.70
TransUnet	105.32M	38.52G	77.48	31.69
SwinUnet	**27.17M**	**6.20G**	79.13	21.55
PVT-CASCADE	*35.27M*	*8.20G*	81.06	20.23
MISSFormer	42.46M	54.46G	81.96	18.20
ScaleFormer(Huang)	111.60M	48.93G	82.86	16.81
DAEFormer	48.07M	27.88G	82.63	17.46
MAXFormer	88.93M	43.85G	*83.66*	*15.89*
ScaleFormer-CASCADE (Ours)	35.84M	10.20G	82.76	16.82
ScaleFormer (ours)	69.00M	40.72G	**84**.**00**	**12**.**30**

**Fig. 7:**
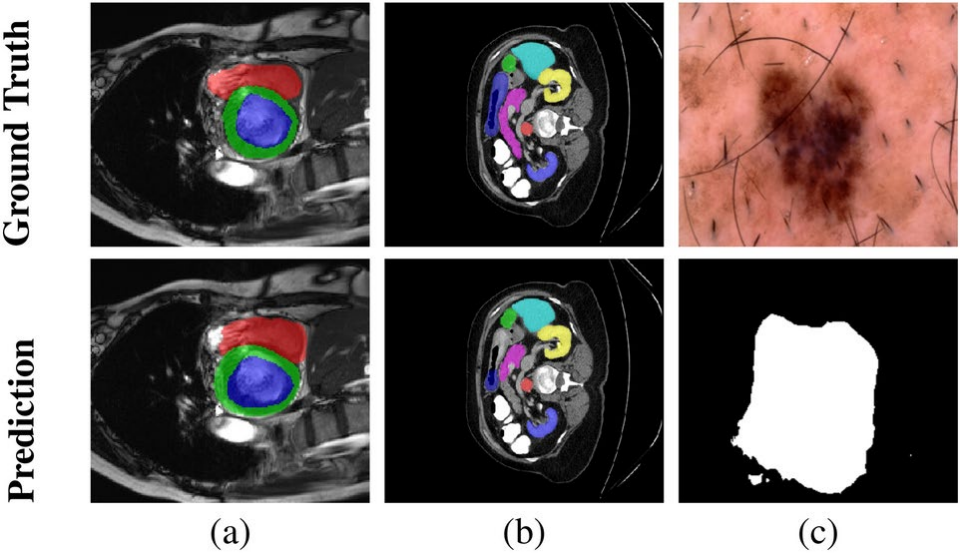
Examples of failure cases in segmentation.

Beyond computational efficiency, our method also encounters challenges in accurately delineating boundaries, particularly in regions with low contrast transitions. As observed in the ACDC dataset (Fig. 7a), the segmentation of the myocardium and right ventricle often exhibits deviations from the ground truth, primarily due to the gradual intensity variations between adjacent structures. Similarly, in the Synapse dataset (Fig. 7b), the pancreas is frequently misclassified due to their similar intensity distributions with surrounding tissues, making differentiation difficult. In the ISIC2018 dataset (Fig. 7c), segmentation errors occur in cases where skin lesions exhibit irregular textures and heterogeneous pigmentation, leading to over-segmentation or under-segmentation. These findings suggest that while our model improves feature representation, its ability to capture fine-grained details remains limited in challenging anatomical structures and complex imaging conditions. To further enhance the performance and robustness of our method, several improvements could be considered. Integrating edge-aware loss functions may help refine boundary delineation, while adaptive thresholding techniques could improve lesion segmentation in dermatological images. Additionally, Inspired by Umirzakova et al.^50^, leveraging residual feature distillation and channel attention mechanisms could enhance feature representation, leading to more precise segmentation. Future work will focus on incorporating advanced feature learning strategies to improve generalization and segmentation accuracy across diverse datasets.

## Conclusion

In this paper, we introduced a novel Grouped Multi-Scale Attention (GMSA) mechanism to enhance multi-scale feature representation in medical image segmentation. Unlike previous methods that rely on uniform-scale feature maps within a single attention layer, GMSA maintains multi-scale feature maps and applies self-attention at various scales by grouping channels and assigning different attention operations to each group. Furthermore, we proposed the Inter-Scale Attention (ISA) mechanism to facilitate cross-scale information exchange, leveraging a combination of channel-aware attention and global pooling to improve feature interaction. To further refine the segmentation process, we designed a feature-rich decoder incorporating the Context Perception Block (CPB), which integrates channel-space compression and spatial gating for effective spatial-channel fusion. Additionally, the Inception module enables richer multi-scale feature extraction, while spatial and channel attention mechanisms emphasize critical structural information. Extensive experiments on the Synapse, ACDC, and ISIC2018 datasets demonstrate that our model effectively handles medical images with diverse structural, textural, and morphological variations, achieving competitive segmentation performance. While our method achieves strong performance, there is room for further improvement in computational efficiency and robustness, as discussed in “Discussion”. Future research could explore more lightweight designs, enhanced boundary refinement techniques, and adaptive feature learning strategies to further optimize model performance and generalization.

## Data Availability

The datasets used in this study are open-source and publicly available. The Synapse Dataset can be accessed at [https://www.synapse.org/Synapse:syn3193805/wiki/89480], the ACDC Dataset is available at [https://www.creatis.insa-lyon.fr/Challenge/acdc/databases.html], and the ISIC2018 Dataset can be found at [https://challenge.isic-archive.com/data/]. These datasets were utilized to assess the performance of the proposed model.
